# BALLU2: A Safe and Affordable Buoyancy Assisted Biped

**DOI:** 10.3389/frobt.2021.730323

**Published:** 2021-12-08

**Authors:** Hosik Chae, Min Sung Ahn, Donghun Noh, Hyunwoo Nam, Dennis Hong

**Affiliations:** Robotics and Mechanisms Laboratory (RoMeLa), Department of Mechanical and Aerospace Engineering, University of California, Los Angeles (UCLA), Los Angeles, CA, United States

**Keywords:** bipedal locomotion, data-driven control, nonlinear modeling, dimension reduction, machine learning, underactuated system, low-cost robot, robot safety

## Abstract

This work presents the first full disclosure of BALLU, Buoyancy Assisted Lightweight Legged Unit, and describes the advantages and challenges of its concept, the hardware design of a new implementation (BALLU2), a motion analysis, and a data-driven walking controller. BALLU is a robot that never falls down due to the buoyancy provided by a set of helium balloons attached to the lightweight body, which solves many issues that hinder current robots from operating close to humans. The advantages gained also lead to the platform’s distinct difficulties caused by severe nonlinearities and external forces such as buoyancy and drag. The paper describes the nonconventional characteristics of BALLU as a legged robot and then gives an analysis of its unique behavior. Based on the analysis, a data-driven approach is proposed to achieve non-teleoperated walking: a statistical process using Spearman Correlation Coefficient is proposed to form low-dimensional state vectors from the simulation data, and an artificial neural network-based controller is trained on the same data. The controller is tested both on simulation and on real-world hardware. Its performance is assessed by observing the robot’s limit cycles and trajectories in the Cartesian coordinate. The controller generates periodic walking sequences in simulation as well as on the real-world robot even without additional transfer learning. It is also shown that the controller can deal with unseen conditions during the training phase. The resulting behavior not only shows the robustness of the controller but also implies that the proposed statistical process effectively extracts a state vector that is low-dimensional yet contains the essential information of the high-dimensional dynamics of BALLU’s walking.

## 1 Introduction

One class of robots most commonly used in our daily lives is service robots. In particular, a central application is social robots that interact with humans and provide information. For instance, LG Electronics’ CLOi at an airport (Incheon Airport, 2019) leads passengers to find a route inside the airport and informs them of their flight schedule. SoftBank’s Pepper at a library (Roanoke County Public Library, 2018) helps the visitors find books. As a similar example, LinkedIn makes the use of Double Robotics’ telepresence robot to telecommute (Double Robotics, 2015). These robots are more economical than human employees in providing an intuitive and easy interface to information. As a result, more service providers are seeking to introduce robots to their business (Aymerich-Franch and Ferrer, 2020; Fukawa, 2020).

Meanwhile, these service robots are all wheeled robots, and there are only a few legged ones such as LARA (Ahn et al., 2019b) and Connie (IBM, 2016). However, though these legged service robots can provide information standing on a reception desk, they are small so limited in taking advantage of mobility as legged robots. On the other hand, most of the legged robots near human size pursue to be strong and powerful, and Boston Dynamics’ Spot (Boston Dynamics, 2019) and ANYbotics’ ANYmal (ANYbotics, 2021) would fall into this case. Taking advantage of the characteristics, they are preferred by some specialized industries such as last-mile delivery or construction site inspection.

While these platforms have shown remarkable progress in technology, essential yet often overlooked aspects that are contributing to their full deployment in close proximity to humans are safety and cost. When they malfunction, the heavyweight and powerful actuation methods could act as a potential cause of serious damage to its surrounding environments and even threaten human lives. In the context of service robots, such robots’ capacity might be redundant for common needs in everyday life but also dangerous.

Additionally, these machines are still expensive for households to adopt. Since many conventional robots use powerful and strong components, they are much higher priced than your average home appliances, and the most affordable and small-scaled quadruped is 2700 USD at the time of this contribution (Unitree, 2021).

Made with helium balloons and lightweight body parts, BALLU (Buoyancy Assisted Lightweight Legged Unit) has the possibility to overcome the aforementioned issues concerning existing robots. The first is safety and inherent stability. Because of the buoyancy provided by the balloons, BALLU is a robot that literally cannot fall down. More importantly, its light parts and soft balloon body can only generate so much momentum and force, allowing it to be operated without worry when there is physical interaction or even collision with young children. This allows BALLU to potentially act in the future as a safe, interactive service robot in the vicinity of people. The second is its cost. BALLU is merely as affordable as many low-cost home appliances. In the long run, this even opens up opportunities for such platforms to act as disposable robots, where a number of them can be easily built and explore unknown environments with the less economic burden.

Since the concept of BALLU was first unveiled by Ghassemi and Hong (2016), there have been further studies of robot platforms adopting helium balloons and leveraging their buoyancy force. One noticeable work is the Giacometti Arm designed by Takeichi et al. (2017), a 20-m helium balloon supported robot arm designed for inspection tasks. This manipulator has 20 joints driven by pneumatic and thin artificial muscles. Among mobile platforms, GerWalk by Yamada and Nakamura (2018) is one that is very resemblant to BALLU. Because its body is a helium balloon, it is able to easily traverse stairs and other obstacles with stability. Nayar et al. (2019) of JPL also proposed a balloon based walking robot for Mars exploration.

Unlike the previously mentioned platforms that rely on passively acting forces (e.g., buoyancy in the case with helium balloons), there are also works that have directly integrated active thrusters. The concept of a bipedal robot supported by a propulsion system is first proposed by Zhang et al. (2016). Aerial-biped by Maekawa et al. (2018) is a bipedal robot attached to a quadrotor, and the robot walks using a gait sequence generated by a policy learned by reinforcement learning. Though it is not published, LEONARDO from Aerospace Robotics and Control at Caltech (2019) is another bipedal robot with drone-like propellers. On a more extreme note, the feasibility of a combination of propellers, buoyancy force, and active rappelling to lift rigid bodies has also been studied by Lin et al. (2019).

Ghassemi and Hong (2016) introduced the first iteration of BALLU to verify the concept of buoyancy assisted legged robot, but it was limited to teleoperation using radio control signals. In this paper, the next iteration, BALLU2, which is the first implementation that can walk by algorithms, is presented with details on its design and controller. The contributions of this work are as follows:

• We first disclose in detail the concept, advantages, and challenges of the BALLU platform, a buoyancy assisted bipedal robot that is affordable and safe.
• We improved the platform and built a new version, BALLU2, which has a simpler and more robust design. The onboard computer newly adopted allows BALLU2 to be controlled by an algorithm.
• Based on an analysis of BALLU’s motion, we proposed the first data-driven walking algorithm for BALLU. The proposed approach is comprised of the following two components:
◦ We presented a statistical method that can extract a state vector from data collected from the highly nonlinear dynamics, using Spearman Correlation Coefficient. The state vector is low dimensional yet contains essential information of the high dimensional system.
◦ We trained a planar data-driven walking controller with artificial neural networks using the definition of the state and a set of expert data.

The remaining part of the paper is organized as follows: Starting with hardware design and software architecture, and mathematical modeling are described in Section 2. Section 3 explains the platform’s unique challenges and describes BALLU’s distinct behaviors during locomotion per the authors’ experiences. Section 4 proposes the first non-remote controlled walking strategy for BALLU, and Section 5 and Section 6 shows the experiment setup and the results and insights. Finally, Section 7 concludes the paper.

## 2 System Description

### 2.1 Hardware Design

**FIGURE 1:**
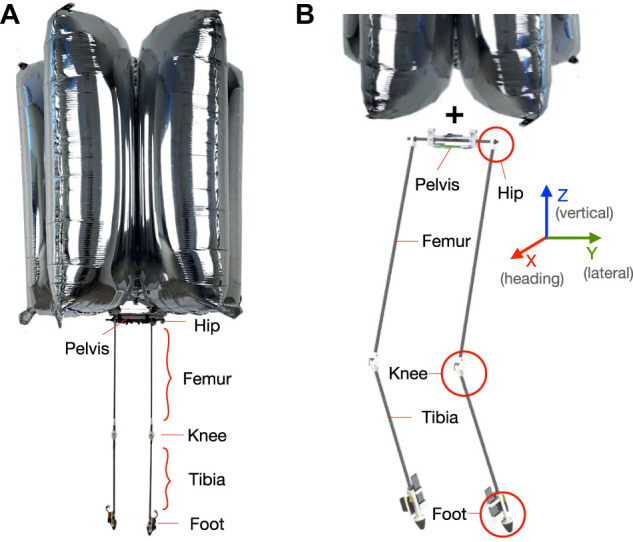
The overall design of BALLU and the name of each part.

At its core, BALLU is a bipedal robot attached to a set of helium balloons, which provide sufficient buoyancy force to prevent the robot from falling down. As shown in Figure 1, the body consists of a pelvis link, which has two identical legs attached to its ends, with each leg having a joint at the hip and the knee. To reduce the weight that the buoyancy has to support, the majority of the components are made or chosen to be light, with unavoidably heavy components being placed at the foot. The detailed design parameters are listed in Table 1.

**TABLE 1:** Design parameters.

	Symbol	Value	Description
Mass [g]	*m*_*p*_	31.2	Pelvis link with electronics
	*m*_*fm*_	9.0	Femur link
	*m*_*tb*_	9.2	Tibia link
	*m*_*hp*_	2.2	Hip joint
	*m*_*kn*_	6.1	Knee joint
	*m*_*ft*_	23.5	Foot part with electronics
	*m*_*balloon*_	19.8	Single balloon
Length [mm]	*l*_*pelvis*_	163.0	Pelvis
	*l*_*femur*_	370.0	Femur
	*l*_*tibia*_	385.0	Tibia
	*l*_*fm*_	185.0	From Knee to Femur’s center
	*l*_*tb*_	192.5	From Knee to Tibia’s center
	*l*_*ft*_	370.0	From Knee to Foot’s center
Force [gf]	*F*_*B*_	195.0	Buoyanct force due to helium gas
	*F*_*b*_	76.2	Net buoyancy

In BALLU2, to enhance BALLU’s functionality, several changes have been made to the BALLU1’s design. The new iteration of BALLU can primarily be broken up into four components: the balloons, the pelvis link, the leg, the knee joint, and the foot.

#### 2.1.1 Balloons

Because the buoyancy force plays an important role for BALLU, such a consistent and reliable external force is achieved through the use of off-the-shelf mylar balloons filled with helium. These balloons are low-cost and easy to purchase, while they also result in minimal deflation per the authors’ experience. Any number of balloons (in our case, we have experimented with 2–6) with various shapes could be used, and they are held together by threading lightweight wires through holes located at the balloon’s corners.

The magnitude of the balloons’ net buoyancy, which is the difference between the buoyancy of the helium and the weight of the balloons, must be smaller than the total body weight to prevent the robot from floating in the air. This net buoyancy is controlled to support most of the body weight, with the normal forces at each foot supporting the rest.

In practice, as injecting the same amount of helium every time to keep the body afloat is difficult, a generous amount of helium is initially injected, and counterweights are attached to the body to adjust the net upward force. This is done by calibrating the robot’s normal weight on a scale, which is empirically chosen to be 55 gf in the presented version of BALLU. Such a choice allows the robot to stay vertically upright when in a double support phase, but sink when in a single support phase, which will be important in the subsequent locomotion approach.

#### 2.1.2 Pelvis Link

The pelvis link is what holds the legs and the balloons together, and is also the mount for BALLU’s onboard controller. A Raspberry Pi Zero W is used for its low-cost, lightweight, potential onboard computing, and flexible communication (e.g. WiFi and Bluetooth) capabilities. These updates are a distinct difference from the previous version (Ghassemi and Hong, 2016), which was limited to teleoperation via radio controllers, and allows BALLU2 to walk based on algorithms.

In control aspects, it is a convenient choice to take the center of the pelvis link as the origin of the floating body since the pelvis is where all the forces from the legs and the balloons are congregated at.

#### 2.1.3 Legs

Each leg is a modular component that can be attached to the pelvis link, comprised of a hip joint, a femur and tibia links, a knee joint holding the two links, and a foot. The hip joint is simply a 3D printed part with a bearing in it that slots around the pelvis link so that they freely swing without actuation. The links are hollow and square carbon fiber parts that the wires go through. The knee joints and feet are presented in the following sections.

#### 2.1.4 Knee Joints

**FIGURE 2:**
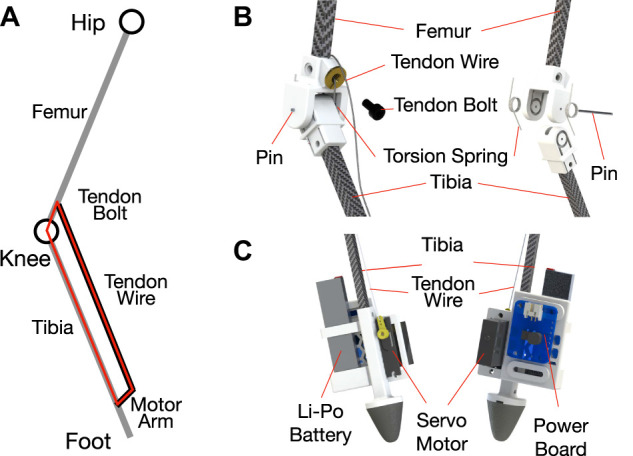
**(A)** Four-bar linkage mechanism. **(B)** Knee joint design. **(C)** Foot Design.

The knee joint design is illustrated in Figure 2B: It consists of upper and lower parts, two symmetric torsion springs attached to each side, a metal pin, a tendon wire, and a tendon bolt. As shown in Figure 2A, the motor arm, tendon adjustment module, the lower femur, and tibia form a four-bar linkage mechanism. The knee springs are preloaded, and it allows for the knee to be quickly unbent when the wire is not in tension. The tendon wire starting from the servo motor arm goes into the socket embedded behind the upper joint part, and the initial knee joint angle and the length of the tendon wire can be adjusted with a bolt.

**FIGURE 3:**
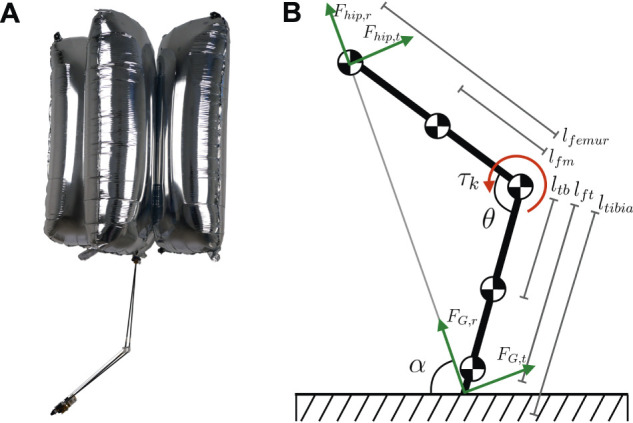
**(A)** An example of sink down. **(B)** A schematic diagram of a leg seen from the side.

The spring constant is determined so that the legs extend in a double support phase and bend in a single support phase, which assists with controlling BALLU, as will be described in Sections 3.3 and Section 3.4. Considering Figure 3B, these conditions can be represented first in terms of the vertical force acting on the pelvis

$$\begin{array}{ll} F_{p,z,ds} & =2F-m_{p}g+F_{b}>0 \end{array}$$ (1)

$$\begin{array}{ll} F_{p,z,ss} & =F-\left(m_{p}+m_{leg}\right)g+F_{b}<0 \end{array}$$ (2)

 where

$$\begin{array}{ll} m_{leg} & =m_{hp}+m_{fr}+m_{kn}+m_{tb}+m_{ft} \end{array}$$ (3)

$$\begin{array}{ll} F & =F_{hip,t}\cos\alpha +F_{hip,r}\sin\alpha \end{array}$$ (4)

$$\begin{array}{ll} F_{hip,r} & =A\left(\theta ,l\right)\left(l_{fm}+l_{tb}\right)\tau_{k}+f_{hip,r}\left(\theta ,\alpha ,l,m\right) \end{array}$$ (5)

$$\begin{array}{ll} F_{hip,t} & =B\left(\theta ,l\right)\left(l_{fm}-l_{tb}\right)\tau_{k}+f_{hip,t}\left(\theta ,\alpha ,l,m\right) \end{array}$$ (6)

*F*_*hip*,*r*_ is the force from the leg at the hip in the radial direction connecting the hip and the foot, and *F*_*hip*,*t*_ is in the tangential direction.

The inequalities Eqs 1, 2 can be rewritten in terms of the knee spring torque. The torsion spring is preloaded, and the torque should be positive since we only want to consider the torque in straightening the knee joint.

$$\begin{array}{ll} & \tau_{k}=\kappa \left(\theta +\theta_{0}\right) \end{array}$$ (7)

$$\begin{array}{ll} \tau_{k,ds}< & \tau_{k}<\tau_{k,ss} \end{array}$$ (8)

$$\begin{array}{ll} & \tau_{k}>0 \end{array}$$ (9)

For the design parameters and the knee joint displacement *θ* within the joint limits, a range of spring constant *κ* can be obtained from Eq. 8. Among satisfying *κ*, we prefer to choose a relatively low stiffness. It is because, when the spring constant takes a lower value, the dynamics change more drastically between a single support phase and a double support phase, and the controller would have more options to control its motion by adjusting each phase length. Through a handful of empirical tests, a torsion spring with 0.1409 N mm/deg with 135° was chosen.

#### 2.1.5 Feet

Unlike the relatively lightweight legs, the feet hold heavier components, which include a power board (Adafruit PowerBoost 1000 Basic), a 3.7 V Lithium Polymer battery, and a servo motor (Dymond D47). The power board converts 3.7–5 V for the servos and the computing board at the pelvis. The computing board commands the servo, which effectively actuates the knee through a wire-driven four-bar mechanism as shown in Figure 2C. For high friction point-like contact, a cone-shaped rubber is attached at the end.

### 2.2 System Architecture

The current system is set up for easy and modular development, as various processes are concurrently running on board, including the motor controller and the communication and messaging module. Shared memory is leveraged to share data between processes. Python is primarily used for simplicity, while C++ is used for low-level modules.

### 2.3 Simulation Environment

Coppelia Robotics’ CoppeliaSim Rohmer et al. (2013) is used for simulation, and a few custom add-ons were developed to better capture BALLU’s unique characteristics. Particularly important to BALLU was the support for aerodynamic forces, specifically buoyancy and drag.

#### 2.3.1 Buoyancy

An external force that always acts in the opposite direction of gravity is applied to the robot’s pelvis link. The magnitude of this net buoyant force is calibrated for the physical platform to maintain a normal force of 55 gf.

#### 2.3.2 Drag

What is just as important as buoyancy for BALLU is a drag. Because the robot is lightweight yet the body takes a large portion, drag force plays a nontrivial role in BALLU’s orientation. Consequently, the drag forces for the transitional and rotational directions with the robot’s X, Y, and Z-axes were calculated using computational fluid dynamics software. Because the lateral distance between two feet keeps the robot from rotating in the roll direction, only the rotations in the pitch and yaw direction are taken as the domain variables for the computation. Considering the rate of change in the pitch and yaw directions that BALLU normally takes, the drag force was computed over ±40 deg for the pitch and ±5 deg for the yaw angle with the unit speed.

**FIGURE 4:**
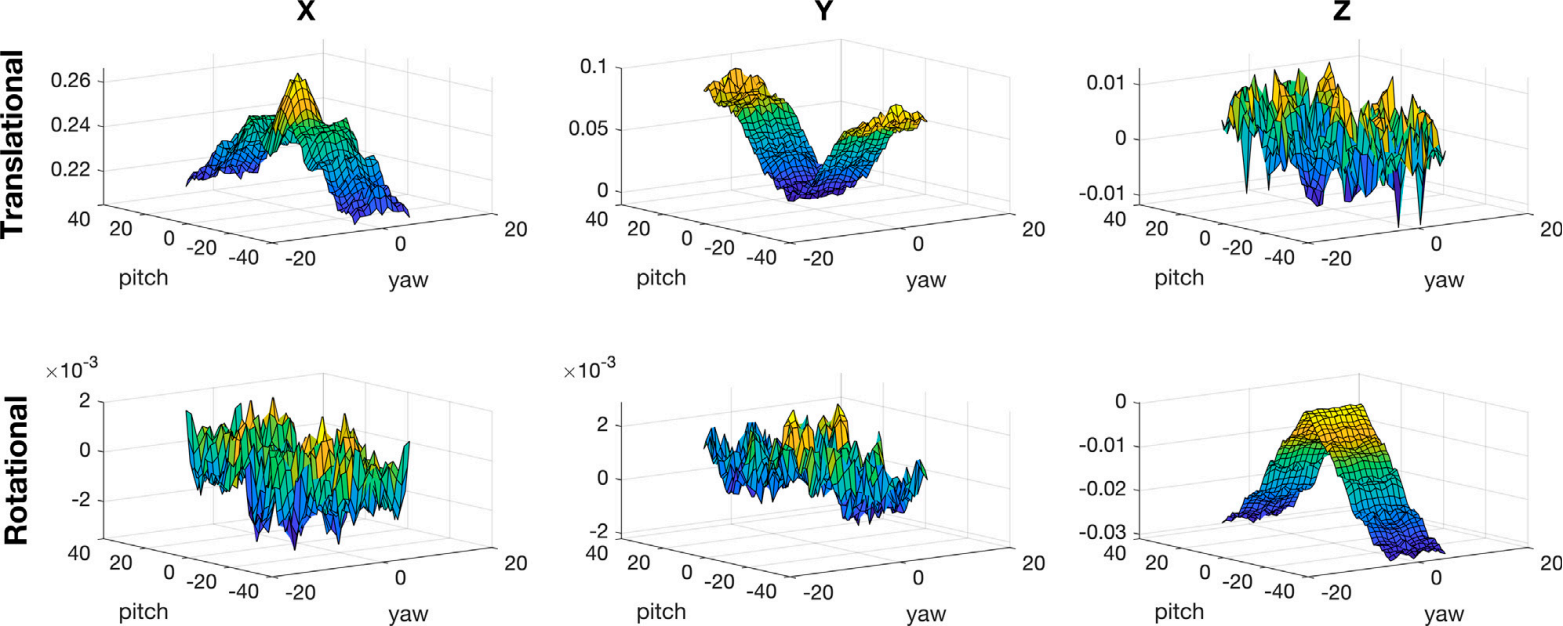
Drag force computed using computational fluid dynamics software. Since they are based on airflow with unit velocity, the drag coefficient and drag force can be calculated using them. The X-axis directs the heading direction, the Y-axis lateral, and the Z-axis vertical.

The results represented in Figure 4 show that the translational force in the *X* (heading)-axis was dominant (7.0 ∼ 1000.0 times larger) among the remaining five directions. Although the value in the *Y*-direction might be comparable, again, the robot barely moves in the lateral direction due to the distance between the two feet, and the expected lateral drag force is very small. It suggests that it would be sufficient to model the drag force as a single force acting in the *X*-direction.

We adopt the following commonly used model for representing transitional drag force, which is a quadratic function of the relative flow speed of the object to the fluid:

$$F_{drag}=-\frac{1}{2}C_{d}\rho Av_{com}^{2}$$ (10)

where *C*_*d*_ is the drag coefficient, *A* is the reference area which is a function of the heading direction, and *v*_*com*_ is the speed of the robot.

The simulation result was taken as the drag coefficient at a pitch and yaw pair since it is computed with unit speed. In the dynamics simulation, the drag force is computed using the model and applied as an opposing force in the simulation.

## 3 Motion Analysis

BALLU is a unique bipedal robot that, because of the external buoyancy force and its simple configuration, exhibits a distinct locomotion behavior different from conventional bipeds. When a first-time operator is tasked to trigger each leg individually and make the robot walk, it can respond with non-intuitive behaviors. The most noticeable is the turning in the yaw direction, where because of the underactuation and the mass of the legs relative to the entire body, the body’s yaw orientation can change significantly depending on the duration that the robot is in the single stance phase. Furthermore, as the body oscillates up and down during locomotion and since buoyancy and drag distort the speed at which the robot moves, BALLU looks as if it is slowly striding in space. Such non-intuitive and unconventional behavior inherent by the design calls for a detailed analysis of the platform’s motions to potentially leverage them for control.

### 3.1 Challenge

BALLU is a robot that never falls down. It is a fact that BALLU, by design, cannot damage its surrounding environment or itself, unlike other heavier robots. However, interestingly, BALLU has its own counterpart to a conventional biped’s “fall” state, which is called “sink down” state. In this state, the robot has *sunk down* as shown in Figure 3A. BALLU’s body is slightly heavier than the net buoyancy that the balloons can exert. Hence, without any control, it sinks down until reaching equilibrium between the buoyancy, the GRFs, and the straightening force from the compressed knee joints. In this state, it is difficult to conduct any meaningful motion. In the sink down state, the knee joints are close to the joint limits and it is hard to make the leg swing without dragging its foot, despite most of the body parts still floating. Therefore, it is necessary for BALLU to manage its state with properly coordinated walking motions and avoid sinking down.

#### 3.1.1 Underactuation

BALLU has only 2 DoF for each leg and only 1 active DoF on the knee because the hip joint is passive and freely rotates. Unlike the majority of robots that can follow the desired trajectory generated by a controller, passive dynamics govern BALLU’s hip joints and the controller has to realize the desired motion considering that the hip joint can only swing freely.

#### 3.1.2 Nonlinear Dynamics

The drag force disrupts BALLU’s walking motion because of the balloon’s large cross-section. This adds additional complexity to the already nonlinear dynamics of the rigid body. The large fluctuation makes it even harder to analyze. The magnitude is about 5.5% compared to its body weight and can peak up to about 12.0%. Moreover, there are multiple sources of uncertainty on the platform. For example, while it is necessary to adopt lightweight and affordable parts, smaller and low-cost sensors and actuators tend to have lower fidelity. In addition, light materials are prone to wear, and helium balloons lose their buoyancy over time, which makes the system time-varying and the identified parameters unknown.

Developing a motion planner for BALLU is a nontrivial task. From the authors’ remote control experiences, BALLU is able to walk, climb stairs, jump, and turn with proper actuation timing. However, because of the complex interaction between the balloons, which are affected by aerodynamics, and the underactuated rigid body, it is difficult and counter-intuitive to imagine how BALLU should locomote. To find insights from observing behaviors from successful teleoperations, a substantial amount of remote control experiments in various environments were done. As a result, the authors were able to get a few insights that would be the cornerstones to develop a controller in Section 4 and the future.

### 3.2 Spring Force Direction on Body

Figure 3B depicts the side view of a leg. Decomposed into the radial direction connecting the hip and the foot and the tangential direction, the force applied to the pelvis link through the hip joint is represented as Eqs 5, 6, where fhip;r and fhip;t are the rest of the terms not related to the spring torque of the knee joint, and they are mostly gravitational effects. Since the differences between lfm and ltb are negligible, the spring torque from the knee joint only contributes as a radial force.

This means the leg pushes off the pelvis along the line connecting the foot and the pelvis and allows to look at the entire body dynamics as interactions between the pelvis link and the two force vectors from each leg.

### 3.3 Height Control Strategy

Continuing the discussion in the previous section, we can analyze the relationship between the foot positions and the height of the body. In particular, the height shows a different pattern in single support phases and double support phases.

#### 3.3.1 Double Support

**FIGURE 5:**
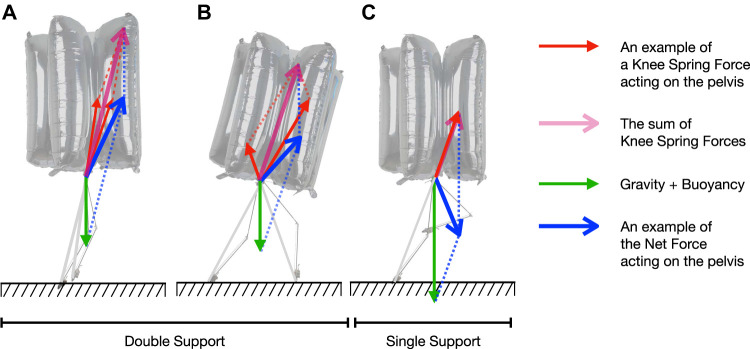
The forces acting on the pelvis and the potential resultant force: **(A)** Double support phase (two feet are close), **(B)** Double support phase (two feet are distant), and **(C)** Single support phase. In a double support phase, the robot is expected to gain positive vertical acceleration. In a single support phase, it is expected to gain positive forward acceleration and negative vertical acceleration.

As shown in Figure 5B and Figure 5A, the pelvis is supported by two forces, and the resultant force acting on the pelvis link is more likely to be upward. In particular, when the footsteps are close, the forces are more focused in the vertical direction and strongly push the pelvis link (Figure 5A). When the two footsteps are far (Figure 5B), the front leg’s knee is almost relaxed and the contribution from the hind leg is greater. The hind leg pushes the pelvis so that it moves towards the front and upward direction.

#### 3.3.2 Single Support

When the robot is in a single support phase (Figure 5C), there is only one supporting force upward, and additionally, the force needs to support the weight of the swing leg, resulting in the height decreasing, and the body sinking down.

This behavior suggests that some feedback controllers could potentially regulate the height of the pelvis by looking at the current height and allocate appropriate single support and double support phases.

### 3.4 Speed Regulation Strategy

During remote control, there are a few ways to control the forward speed. One way is to control the swing time. Since a leg takes a great part of BALLU’s total weight, after passing the nadir of its swing, the whole body gains velocity from the moment of the swing leg. The closer the leg swings to the apex, the greater forward speed the body gains when it exits the single support phase. However, the length of a single support phase directly affects the following footstep position, which influences the states in the subsequent phases.

If BALLU’s feet are behind the center of the pelvis during the double support phase, the horizontal components of the two legs are effectively aligned and the robot can induce a large acceleration. However, this has a risk, as the robot can fall into the aforementioned sink-down state if it holds this state for too long.

When BALLU’s speed drops considerably, it can recover it through a large footstep. As mentioned in Section 3.5, if it can position both feet forward, the further the feet are put, the larger acceleration the body gains when it reaches the apex. During this sequence, BALLU can recover both height and velocity, and move on to the next sequence of motions.

### 3.5 Footstep Position Selection

One widely used method Hooks et al. (2020), Ahn and Hong (2020), Bledt et al. (2018) known as Raibert heuristics Raibert (1986) is often used in legged robots to determine the next footstep position:

$$x_{f}=\frac{1}{2}\dot{x}T_{s}+k_{\dot{x}}\left(\dot{x}-{\dot{x}}_{des}\right)$$ (11)

where *x*_*f*_ is the next footstep position with respect to the center of mass, *T*_*s*_ is the duration of the phase, and $\dot{x}$ is the forward speed. The heuristic assumes the robot as a linear inverted pendulum and regulates the robot’s CoM velocity by its foot placement. If the second term in Eq. 11 is positive, the robot steps further than the nominal footstep position and accelerates in the following phase, and if the second term is negative, it steps closer and decelerates.

When BALLU is in a double support phase, the net force acting on the body is upward (unlike a convention robot, whose force would be downwards due to gravity), which results in behaviors that would match those exhibited by Eq. 11, except with a negative on the velocity gain, as shown below:

$$x_{f}=\frac{1}{2}\dot{x}T_{s}-k_{\dot{x}}\left(\dot{x}-{\dot{x}}_{des}\right)$$ (12)

## 4 Proposed Walking Approach

**FIGURE 6:**
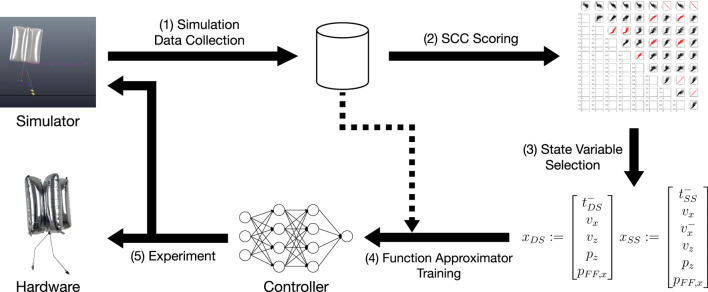
A summary of the proposed approach.

This chapter outlines a preliminary walking algorithm for BALLU2. There are various atypical components in BALLU’s dynamics, and as no other bipeds experience such a situation, it necessitates a different type of walking controller. In this work, we focus on planar walking in the sagittal plane. At a high level, the data-driven approach attempts to extract low dimensional yet essential information that heavily affects a successful walking behavior, out of the numerous observable high-dimensional states. The summary of the proposed approach is demonstrated in Figure 6.

As discussed in the previous sections, it is advantageous to apply different strategies depending on whether the robot is in a single or a double support phase. Moreover, if the actuation profile is fixed, the phase time becomes the only parameter that determines the walking motion in each phase. This relationship is given by

$$\begin{array}{cc} x_{DS}^{out} & =f_{DS}\left(t_{DS},x_{DS}^{in}\right)\\ x_{SS}^{out} & =f_{SS}\left(t_{SS},x_{SS}^{in}\right) \end{array}$$ (13)

where *f*_*DS*_ and *f*_*SS*_ are transition functions for each phase, and the “state” *x* ∈ X , where X is the state space. For clarification, the term “state” is loosely used in this work to represent any potential variables pertaining to the robot during its locomotion. To constrain the actuation profile, it is assumed that the motors accept only the binary input and instantly move to each extreme position.

Rather than directly finding each transition function, the proposed approach looks for an inverse relationship between *x* ^*out*^ and t_*_, *g*, and its approximation $\hat{g}$ from data as follows:

$$\begin{array}{cc} t_{DS} & \approx \hat{g}DS\left(x^{out},x^{in}\right)\\ t_{SS} & \approx \hat{g}SS\left(x^{out},x^{in}\right) \end{array}$$ (14)

### 4.1 Data Correlation Investigation

To determine which variables should be considered in the state vector, a statistical investigation was first conducted on the extensive potential relationships between the state variables. Spearman Correlation Coefficient (SCC) was used to account for the nonlinearities in the variables’ relationships as well as the indicator’s simplicity.

The analysis in Section 3 suggests the possibility that the BALLU’s walking dynamics can be written in terms of kinematic quantities and relationships between them, and the potential state variables are listed based on this assumption. Table 2 is the full list of the potential states investigated. For each variable, not only were the correlations between the different types of temporally adjacent phases (e.g. single support and double support) considered, but also between the same type of phases (e.g. double phase to double phase). SCCs were calculated for all variables, and only those larger than a chosen threshold of 0.85 are taken. Note that the state can drastically change within each phase, which could result in two very different states upon entering and exiting a phase. Consequently, the states’ correlations are evaluated at both the beginning and end of a phase. During implementation, the state upon entering a phase will be determined by the sensor signals and the output will be the desired states.

**TABLE 2:** Potential state variables.

Variable	Description
*T*_*phase*_	The time duration of a phase, *phase* ∈{*DS*, *SS*}
*t*_*act*,*L*_, *t*_*act*,*R*_	The duration of actuation (left, right)
*p*_*c*,*x*_, *p*_*c*,*z*_	The position of the center of mass of the pelvis
*v*_*c*,*x*_, *v*_*c*,*z*_	The velocity of center of mass of the pelvis
*p*_*b*,*x*_	The position of balloon
*v*_*b*,*x*_	The velocity of balloon
*α*_*p*_, *β*_*p*_, *γ*_*p*_	Orientation of the pelvis about each axes
*s*	Foot distance
*p*_*FF*,*x*_, *p*_*FF*,*z*_	Coordinates of the position of the front foot
*p*_*HF*,*x*_, *p*_*HF*,*z*_	Coordinates of the position of the hind foot
*q*_*n*_, *q*_*h*_, *q*_*k*_, *q*_*m*_	Joint positions of neck, hip, knee, motor
*ω*_*n*_, *ω*_*h*_, *ω*_*k*_, *ω*_*m*_	Joint velocities of neck, hip, knee, motor

All data for analysis were collected in simulation. Not only was nominal walking data from teleoperation collected, but walking sequences that include intentionally elongated and shortened double and single support phases as well as following recovery from such abnormal timings were also recorded.

From multiple simulation trials running at 100 Hz, 149,291 raw data samples were collected, and 1,585 phase changes (740 phase changes into the double support phase and 745 phase changes into the single support phase) were obtained. Each data point contains the variables listed in Table 2. Except for time variables, all variables at the beginning of the phase and those at the end of the phase are paired in each data point.

As a result, the state vectors *x*_*SS*_ and *x*_*DS*_ are $x_{SS}≔{\left[t_{SS}^{-},v_{c,x},v_{c,x}^{-},v_{c,z},p_{c,z},p_{FF,x}\right]}^{T}$ and $x_{DS}≔{\left[t_{DS}^{-},v_{c,x},v_{c,z},p_{c,z},p_{FF,x}\right]}^{T}$ , where the ‘-’ superscript stands for the value of the previous phase. Considering the analysis in Section 3.2, the result is an acceptable choice and consistent with the author’s experience.

### 4.2 Function Approximation

A neural network is trained as a function approximator for each *g*. Both networks individually consist of a multilayer perceptron with ELU nonlinear activation functions and ADAM optimizer with MSE (Minimum Square Error) loss and a constant learning rate.

The hyperparameters are chosen via grid search to have the least test loss. The list of searched hyperparameters and their candidates are the number of hidden layers $h_{l}\in \left\{2^{0},2^{1},\cdots \;,2^{7}\right\}$ , hidden units $h_{u}\in \left\{1,2,3,4\right\}$ , and the learning rate $\alpha \in \left\{0.0001,0.0002,0.0005,0.001,\cdots 0.05,0.01\right\}$ . As a result, a network with three hidden layers with eight hidden units, respectively, and a learning rate of 0.002 were used.

The network is trained for 100 epochs with early stopping. For each set of hyperparameters, they are mostly stopped in less than 50 epochs. Dataset was randomly split so that 70% of data is used for training, 15% for validation, 15% for testing, and the training and validation set is shuffled at every epoch.

**FIGURE 7:**
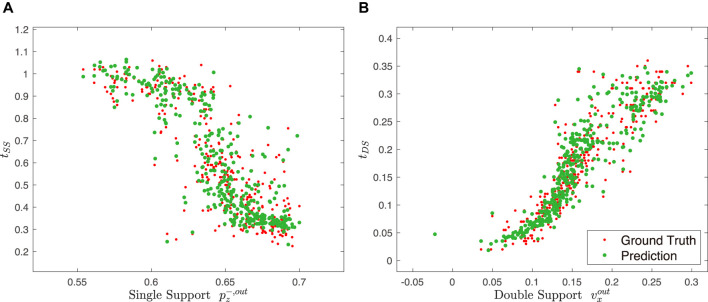
Examples of the results of the training. **(A)** shows the trained relationship between the length of the phase time, *t*_*SS*_, and the *x*-coordinate of the front foot at the beginning of the phase, $p_{z}^{-,out}$ , in the case of a single support phase. **(B)** shows between the phase time, *t*_*DS*_, and the velocity of CoM in *Z*-direction when it leaves the phase, $v_{x}^{out}$ , in the case of a double support phase.

The trained models with the hyperparameters above showed 0.7222 and 0.2177 test errors for the single support model and the double support model. The training result was also qualitatively evaluated, by looking at how well the true data points are covered by the predictions for each variable. For example, Figure 7 shows the relationship between the single support time and the height of the pelvis in the previous single support phase ( $p_{z}^{-,out}$ ), and the double support time and the desired pelvis velocity in *X*-direction at the end of the phase ( $v_{x}^{out}$ ). The prediction by the trained model is widely covering most of the given data.

## 5 Experiment

### 5.1 Simulation

To evaluate how well the proposed approach identifies from data the core states responsible for BALLU’s walking, a set of simulations is conducted not only for walking at a nominal velocity, 0.18 m/s, but also in other varying conditions. By looking at how the controller deals with these variations, not only the robustness of the controller but also whether the statistical approach described in Section 4 has well-extracted variables that are closely related to walking are accessed.

The first set of simulations make changes in mass properties. With heavier feet or pelvis, the body would be easier to sink down. First, the pelvis mass is increased by 6.4% (from 31.2 to 33.2 *g*), and secondly, the feet masses are increased by 16.5% (from 24.2 to 28.2 *g*), respectively. The second set of modifications is the change in commanded velocity, and two simulations are conducted in a slower velocity and a higher velocity.

### 5.2 Hardware Verification

For verification of the proposed locomotion approach, straight walking of about 1.4 m is tested with a desired walking speed of 0.18 m/*s* on the actual hardware.

As BALLU currently does not have an onboard state estimation, the inputs to the neural networks are obtained via color tracking using an off-the-shelf RGBD camera. Using OpenCV and the Intel RealSense D435i, three differently colored LEDs attached to each foot and pelvis are detected. Cartesian coordinates of the colored positions relative to the camera are obtained from RealSense’s internal algorithm, which does include significant noise Ahn et al. (2019a). To mitigate this issue, the coordinates are filtered using a Kalman filter using a constant acceleration model. The data capture and filtering are run at 60 and 100 Hz, respectively, and the positions and velocities obtained from the filter are fed into the trained controller.

## 6 Results and Discussion

### 6.1 Simulation in the Nominal Condition

Because of the complexity and uncertainty of the system, rather than conducting formal nonlinear system analysis, BALLU’s walking performance is first qualitatively assessed by analyzing the pelvis’ trajectories and phase plot.

**FIGURE 8:**
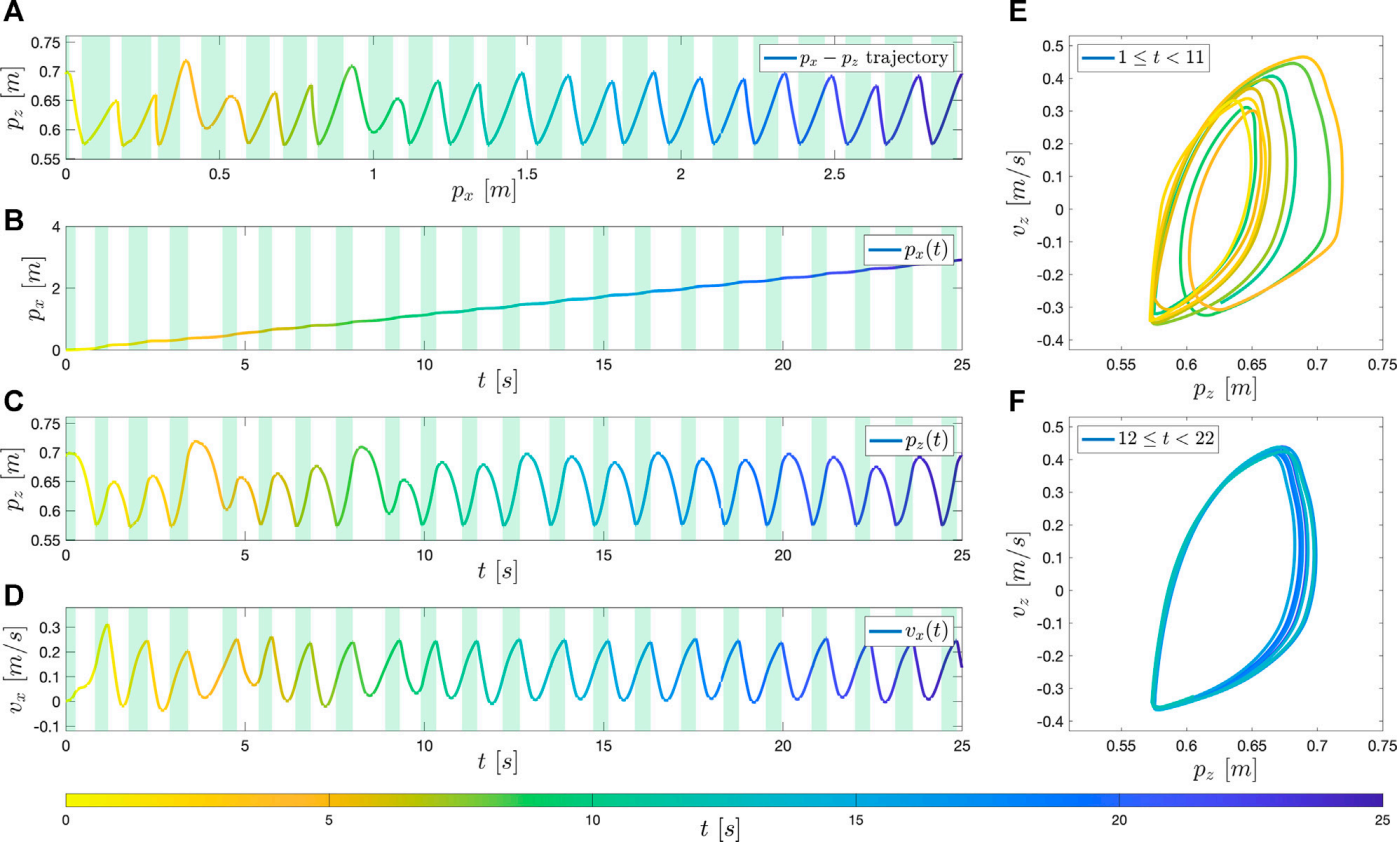
The simulation result in the nominal condition. **(A)** shows the trajectory of the pelvis in the sagittal plane (X-Z plane). **(B–D)** shows the *p*_*x*_, *p*_*z*_, *v*_*x*_ of the pelvis over time. **(E)** and **(F)** are the phase plots of the pelvis in Z-coordinate: **(E)** in an earlier phase (1 s ≤ *t* ≤ 11 s), and **(F)** in a later phase (12 s ≤ *t* ≤ 22 s).

As a baseline, the result in the nominal walking condition is shown in Figures 8A–D. The controller is generating a stable walking sequence. Although the system shows a transient response until around 12 s, a stable and periodic pattern appears since 12 s. As analyzed in Section 3, when it takes single support phases the body height falls and the forward velocity increases, and when the controller takes double support phases (shaded area), the body height rises.

The first noticeable behavior that is important for successful walking is the ability to regulate the body’s height within an interval that BALLU can successfully conduct subsequent motions. In the simulation, we can see that the body height is maintained between 0.58 ∼ 0.70. In the case that such an interval is not preserved, BALLU will exhibit the aforementioned sink-down behavior, leading to an inability to continue walking. Aside from the *Z* height oscillating within an interval, we can also notice that BALLU does indeed stride forward in the *X*-direction and its velocity trajectory shows a gradual increase from rest.

Another interesting point is that the slopes are not symmetric when the pelvis moves up and down and implies BALLU’s unique walking dynamics. It comes from the fact that the velocity in *X*-direction periodically goes up and down. This behavior becomes more obvious later in the hardware test, and the velocity even goes down to the negative. The difference is due to the reality gap including the calibration of the knee joint’s initial position, errors in the mass distribution, and the approximation error of drag force. The corresponding phase plot is shown in Figures 8E,F. Similarly, as BALLU starts from rest, we can observe that the general circular shape of the limit cycle starts small in the earlier state (Figure 8E) but gradually expands until it converges in the latter stage (Figure 8F). This behavior is in parallel with that seen in previous works Ahn and Hong, 2020.

Nonetheless, small fluctuations can be observed in the limit cycle. There can be a couple of explanations for such inconsistency. The first cause is the distribution of the training data. The neural network is trained considering that the expert data is optimal. However, the expert data does not form a perfect limit cycle but, in fact, rather makes a qualitative periodic trajectory. Therefore, it can be expected for the neural net to generate a periodic motion overall as the expert data does but not to make a perfectly overlapped limit cycle.

Another possible explanation can be the neural network’s approximation error. In Figure 7, the ground truth and the predicted values show very close distribution, but there are slight errors between the apparent corresponding pairs. While the neural network outputs the required phase times quite accurately but with a small prediction error, which could contribute to the limit cycle so much out of phase.

Conversely, these two error sources prove the proposed controller’s robustness: the errors do not accumulate, but the controller corrects them and pushes the trajectory back to the limit cycle.

### 6.2 Simulation With Variations

When unexpected changes are given to the normal condition, it was able to observe the controller trying to overcome in the same way that the experts teleoperated, which is analyzed in Section 3.

**FIGURE 9:**
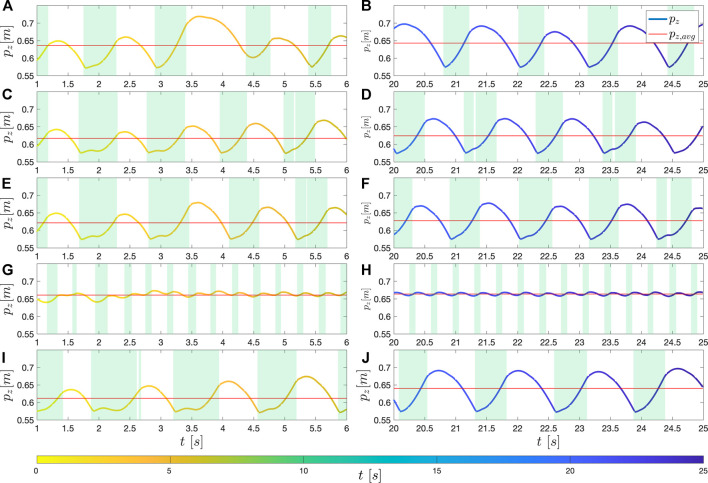
The height of the pelvis, *p*_*z*_, over time when unseen modifications are applied to the model in the nominal condition: **(A,B)** the nominal condition, **(C,D)** increased pelvis mass (+6.4%), **(E,F)** increased feet mass (+165%), **(G,H)** decreased command speed (0.1 m/s), and **(I,J)**: increased command speed (0.32 m/s). The red line is the average height in each time window. The left column is when in an earlier phase (1 s ≤ *t* ≤ 6 s), the right column is when in a latter phase (20 s ≤ *t* ≤ 25 s).

Similar patterns are observed when the pelvis mass increased (Figures 9C,D) and when the feet masses increased (Figures 9E,F). In both cases, the average height is decreased, and the controller takes shorter single support phases and longer double support phases than them in the nominal condition. It can be interpreted that the height of the body falls easier in single support phases because of the increased mass, and the controller tries to regulate the body height not to sink down with a longer double support phase (Section 3.3.1) and minimal single support phase to track the commanded velocity. Since it is difficult to gain speed with the shortened single support phase, they were worse in tracking the desired speed.

In addition, the above results imply that concentrating weight on the feet is more advantageous than putting weight on the pelvis. While those two results show similar pelvis trajectories, the increment on feet is four times larger than that on the pelvis, and the controller fails if the weights are increased further in both cases. This proves once again our design approach to allocate most of the parts on the feet (Section 2.1).

As a second modification, two unachievable velocities were commanded. When the commanded velocity is too low (Figures 9G,H), it can be observed that the controller takes tiny steps. As a result, the two feet become closer and the lines connecting each foot and the pelvis get towards the vertical so to minimize the forward force. It can be interpreted that the controller tries to take the minimum length of single support phases not to increase speed (Section 3.3.2). As the controller drives the body rather upward, the average height is higher than the nominal condition. Contrarily, the controller takes big steps when the commanded velocity is too high (Figures 9I,J). It is to take single support phases to catch up with the high commanded velocity. Since the controller takes single support phases as much as it can, the average body height is lower than that under the nominal condition.

### 6.3 Hardware Test

**FIGURE 10:**
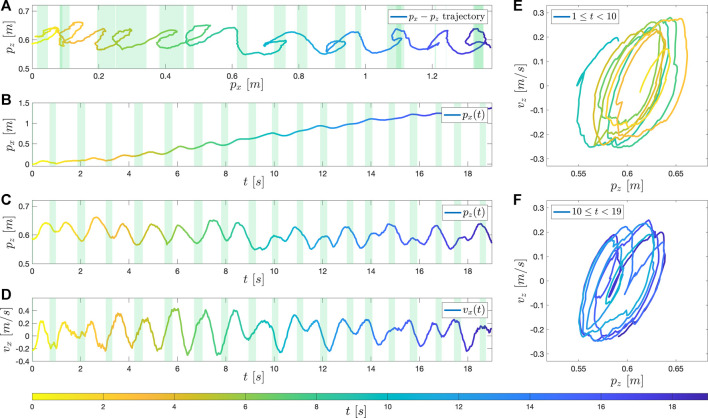
The result on the hardware. **(A)** shows the trajectory of the pelvis in the sagittal plane (X-Z plane). **(B–D)** shows the *p*_*x*_, *p*_*z*_, *v*_*x*_ of the pelvis over time. **(E)** and **(F)** are the phase plots of the pelvis in Z-coordinate: **(E)** in an earlier phase (1 s ≤ *t* ≤ 10 s), and **(F)** in a later phase (10 s ≤ *t* ≤ 19 s).

The trajectories of the pelvis are presented in Figures 10A–D, and the corresponding phase plots are given in Figures 10E,F. Although the response is less uniform and much noisy, the body gradually walks forward in *X*-direction and shows relatively more periodic behavior after 12 *sec*. Considering the significant noise that exists from state estimation, the body height oscillates between 0.54 ∼ 0.66, with an average of approximately 0.6. While the mean may be different, this aligns well with the collected data from the simulation. Specifically, as shown in Figure 10A and Figure 10B, when the height drops [for example, at *p*_*x*_ = 0.19 m (*t* = 4.2 s), *p*_*x*_ = 0.38 m (*t* = 6.65 s), and *p*_*x*_ = 0.61 m (*t* = 8.95 s)] the controller takes longer double support phases to recover the pelvis height. The function approximator, despite being trained based on simulation data, worked well on the physical platform without any additional tuning despite the unavoidable model differences. This suggests that BALLU is capable of walking using the proposed data-driven approach.

In addition, what is more amusing from Figure 10 is BALLU’s negative velocity in the *X*-direction. This behavior is uncommon for bipeds walking forward as the two legs are able to crossover, unlike walking sideways where oscillation is common because the legs cannot crossover. This is a distinct feature of BALLU as walking forward is only achievable by a combination of the support leg’s spring injecting energy into the system and the body and the swing leg’s momentum. Hence, at intervals where such a force and momentum are not sufficient, which includes the period after the swing leg moves past the pelvis (moments after the double support phase in Figure 10), the body temporarily gets pushed backward because of the swing leg moving forward. This behavior is an artifact of the system’s passive dynamics. This reinforces the belief that conventional locomotion controllers may not be suitable for such a system and possibly why the proposed data-driven approach is the first successful non-teleoperated walking for BALLU.

### 6.4 Collision

Touching on safety, it is also evident that BALLU can only produce so much force and momentum in any given direction. In its walking direction, the maximum acceleration and velocity is approximately 0.3 m/*s* ^2^ and 0.4 m/*s*, where the mass of the entire robot is only about 170 *g*. Even then, because of the balloon body’s radius, the first point of collision in a human environment will likely be the balloons and not the legs. This shows that even if a system like BALLU were to malfunction and collide, it will cause no harm to its surrounding environment or humans.

## 7 Conclusion

This work presented the concept and the characteristics of BALLU, analyzed the pattern of its teleoperated walking, and proposed a suitable walking controller. Extending the previous introduction, a new implementation, BALLU2, was developed to be programmatically controllable, and the technical details were disclosed.

By adopting helium balloons and simplifying bipedal locomotion, BALLU is designed to be affordable and lightweight, and these characteristics allow BALLU to be deployed to real-world and service in close proximity to humans. However, the complexity due to aerodynamics and limited actuation introduces another challenge, and it necessitates a nonconventional walking controller.

Based on the careful analysis of the motions from experts’ teleoperation, a data-driven controller is suggested to handle the highly nonlinear dynamics. First, a set of teleoperation data in the simulation were collected in different scenarios. Next, to extract underlying state vectors of the complex dynamics, the correlations between the kinematic quantities were statistically evaluated; for each phase of walking, the quantities that have a high Spearman Correlation Score (SCC) were chosen as state variables. Then, their relationship was approximated using an artificial neural network, which is trained on the same data.

The controller was tested both on simulation and on real-world hardware, and its performances were accessed based on the resulting trajectories and phase plots. In both simulation and hardware experiments, the controller generated stable limit cycles after around 10 s. In particular, the controller worked on the hardware without additional transfer learning. In addition, the controller was tested under unseen conditions during the training condition. The results showed that the robustness of the controller but also suggested that the proposed method was able to extract the key variables that govern BALLU’s walking from the data.

Future work includes expanding the proposed approach to build a walking controller in 3D space as well as an onboard state estimator with more sensors so that walking can be achieved independently. We are also looking forward to applying the proposed statistical method to other nontypical robots and build controllers based on the states extracted from data.

## Data Availability

The original contributions presented in the study are included in the article material, further inquiries can be directed to the corresponding authors.
